# Functionalized Microgel Rods Interlinked into Soft Macroporous Structures for 3D Cell Culture

**DOI:** 10.1002/advs.202103554

**Published:** 2022-01-14

**Authors:** Dirk Rommel, Matthias Mork, Sitara Vedaraman, Céline Bastard, Luis P. B. Guerzoni, Yonca Kittel, Rostislav Vinokur, Nikolai Born, Tamás Haraszti, Laura De Laporte

**Affiliations:** ^1^ DWI – Leibniz Institute for Interactive Materials Aachen 52074 Germany; ^2^ Institute for Technical and Macromolecular Chemistry RWTH Aachen University Aachen 52074 Germany; ^3^ Optics11Life Amsterdam 1081 Netherlands; ^4^ Institute of Applied Medical Engineering Department of Advanced Materials for Biomedicine RWTH Aachen University Aachen 52074 Germany

**Keywords:** 3D cell culture, macroporous microgel scaffolds, microfluidics, rod‐shaped microgels, tissue engineering

## Abstract

In this work, a two component microgel assembly using soft anisometric microgels that interlink to create a 3D macroporous construct for cell growth is reported. Reactive microgel rods with variable aspect ratio are produced via microfluidics in a continuous plug‐flow on‐chip gelation method by photoinitiated free‐radical polymerization of star‐polyethylene glycol‐acrylate with glycidyl methacrylate or 2‐aminoethyl methacrylate comonomers. The resulting complementary epoxy‐ and amine‐functionalized microgels assemble and interlink with each other via a ring opening reaction, resulting in macroporous constructs with pores up to several hundreds of micrometers. The level of crosslinking depends on the functionalization degree of the microgels, which also affects the stiffness and cell adhesiveness of the microgels when modified with the cell‐adhesive GRGDS‐PC peptide. Therefore, 3D spreading and growth of cells inside the macroporous structure is influenced not only by the presence of macropores but also by the mechanical and biochemical properties of the individual microgels.

## Graphical abstract

Two types of microgel rods produced in microfluidics bear active groups enable quick and stable rod interlinking when they come in contact in the aqueous phase. This way, macroporous scaffolds with micro‐ to millimeter‐scale pores are formed that support 3D‐cell culture of human fibroblasts and endothelial cells. Cells attach, spread, and grow, filling the macropores, building biological‐like tissue material.

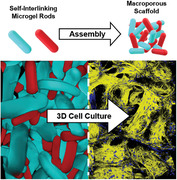

## 1 Introduction

To understand interactions between living matter and artificial materials, tissue engineering research groups focus on the development of 3D scaffolds with increasingly controllable properties, reaching from biochemical interactions at molecular scale to nanoscale hydrogel networks, micrometer‐scale pores, and macroscopic architectures.^[^ ^1^ ^]^ Soft hydrogel scaffolds are conventionally formed by crosslinking a precursor solution, resulting in soft, tissue mimetic constructs. Cells are encapsulated in the precursor before crosslinking, while the formed hydrogel allows for cells to spread and proliferate by degrading and/or remodeling the network.^[^ ^2^ ^]^ As an alternative to bulk hydrogel applications, macroporous 3D scaffolds are fabricated by several techniques like electrospinning, solvent casting, or gas foaming/particle leaching, with the advantage that no degradation is required for cell migration and proliferation, which enhances the rate of cell infiltration.^[^ ^1^, ^3^ ^]^ Control over the porous structure in 3D cell scaffolds is of particular importance as different cell types and their subunits, like neurites, migrate in a limited yet specific manner.^[^ ^3^, ^4^ ^]^

To provide a soft environment with micrometer‐scale pores in contrast to bulk hydrogels or stiff scaffolds, microporous annealed particle (MAP) scaffolds were developed by the Segura group.^[^ ^5^ ^]^ MAPs are injectable and are formed by interlinking microgels together instead of nanoscale precursor molecules, enhancing cell invasion and migration with simultaneous control over the composition of the scaffold building blocks.^[^ ^1^, ^5^, ^6^ ^]^ The macroporous void, referred to as percolated interstitium, can be varied depending on the diameter of the microgels. The position of attached cells to micro‐ and macroporous 3D hydrogel structures in contrast to embedded cells inside bulk‐based 3D hydrogels can provide further insight for proprioception studies to form 3D functional tissue and restore damaged tissue.^[^ ^3^, ^7^ ^]^

In the first MAP report, spherical microgel building blocks were produced via microfluidics by Michael‐type addition, while transglutaminase peptide substrates were coupled into the microgels to interlink them together with activated Factor XIII.^[^ ^5^, ^8^ ^]^ Spherical microgels in the range of 30–150 µm diameter resulted in pore sizes between ≈10 and 45 µm, respectively, which are in a relevant range for cell migration.^[^ ^1^, ^3^, ^4^ ^]^ An increase in microgel stiffness and RGD concentration inside the microgel networks both result in enhanced cell spreading and better proliferation.^[^ ^6a^ ^]^ Cell spreading differences were also observed depending on the microgel dimensions, favoring larger microgels likely due to the larger pore sizes available. Further employment of another biocompatible chemical interlinking mechanism, based on tetrazine norbornene cycloaddition click reaction, reduced inflammation and astrogliosis levels when MAP scaffolds were injected in the brain after a prior initiated photothrombotic stroke.^[^ ^6^, ^9^ ^]^

Alternatively, to covalent bonds between the microgels, supramolecular guest–host (*β*‐cyclodextrin and adamantane) interactions have been employed to interlink complementary functionalized polyethylene glycol (PEG)‐based spherical microgels with diameters ranging from 10 to 100 µm. This led to porous interlinked networks with interparticle distances ranging from ≈5 to 25 µm (after centrifugal filtration) that supported the growth of THP‐1 monocyte cells, which have a migratory phenotype with adhesion‐independent characteristics, enabling the investigation of rapid and dynamic cell behavior at the tissue scale.^[^ ^10^ ^]^

Although this approach resulted in macroporous host–guest networks after being compacted, many microgels with the same functionality were adjacent to each other, without covalently contributing to the stability of the network, compared to complementary functionalized‐alternating microgels that interlink to form a stable 3D scaffold.

In a more rudimentary approach to obtain higher porosity inside injectable hydrogels, mechanical fragmentation has been employed to crush partly crosslinked hydrogels into pieces, which can fuse together. For example, pushing partially crosslinked (2,2,6,6‐tetramethylpiperidin‐1‐yl)oxyl (TEMPO)‐oxidized nanocellulose fibers through a 10 µm mesh resulted in self‐healing of the material into a more porous construct and significantly enhanced cell spreading.^[^ ^11^ ^]^ Another study produced hyaluronan‐methacrylate‐based hydrogel microstrands by extruding a bulk gel through a grid with an aperture size of 40 or 100 µm.^[^ ^12^ ^]^ The initial bulk hydrogel was prepared with or without embedded cells before microstrand formation. This way, two different types of scaffolds could be achieved, comprising cells entrapped between the microstrands outside the gel phase and cells embedded inside the closely entangled gel microstrands. While this fragmentation technology results in direct 3D construct formation, control over the final porosity remains difficult.

Over the last years, the concept or MAPs has been extended to microgel‐based bioinks. PEG‐, agarose‐, and norbornene‐modified hyaluronic acid‐based microgel spheres were prepared via microfluidics and used as jammed, extrudable bioinks for the purpose of 3D bioprinting.^[^ ^13^ ^]^ Here, the macromolecular backbone, crosslinking mechanism, and size of the generated microgel spheres influence the shear‐thinning properties and thus printability of the bioink. The jammed microgel ink showed strain dependent characteristics, yielding at higher strains and demonstrating more elastic behavior at lower strains during 3D bioprinting.

As an alternative to spheres, rod‐ and more complex‐shaped microgels are fabricated via photolithography, in‐mold polymerization, stop‐flow lithography, or microfluidic techniques, allowing for (bio)chemical, physical, and mechanical uniformity utilizing synthetic biocompatible gels.^[^ ^1^, ^14^ ^]^ In an early study, collagen‐based rod‐shaped modules (0.41 mm in diameter and 0.62 mm in length), produced by a multistep technique using in‐tube gelation with subsequent mechanical sectioning and template removal, contained embedded HepG2 cells, while human umbilical vein endothelial cells (HUVECs) were cultured on the surface of the modules. The collagen rods were sequentially placed inside a larger tube to hold the assembly in place.^[^ ^15^ ^]^ The system allowed for perfusion with whole blood due to the resulting macroporosity. While this method uses the anisometry of the modules to obtain larger porosity, the assembly must be maintained by the surrounding container, until sufficient interlinking due to cell proliferation and attachment takes place to stabilize the scaffold. Later, a droplet‐generator microfluidic system was employed to generate 1 to 3 mm long gelatin methacrylate (GelMa) or Matrigel microgel rods containing living cells. These could be arranged into programmed 3D cell culture structures due to subsequent photo‐crosslinking or rapid fusion of rod‐rod interfaces to stabilize the obtained network, respectively.^[^ ^16^ ^]^ Although the fusion of the microgel rods was rapid, this method resulted in lower macroporosity between the rods because they formed predominantly stacked structures.

All these studies demonstrate the relevance and the need of macroporous materials for 3D cell culture. However, to our knowledge, no rod‐based, self‐interlinking synthetic microgel scaffold with a macroporous percolated interstitium in the pore size range of ≈50 to 200 µm has been reported to date.

Here, we report a novel robust manner to create stable micro‐ to millimeter‐scale macroporous constructs by self‐assembly and rapid interlinking of two types of rod‐shaped microgels bearing complementary reactive groups. This method can diversify and enhance the control of porosity of MAP scaffolds using the anisometric microgel dimensions and aspect ratio, while the biochemical and mechanical properties can be altered independently. Compared to spherical microgels‐based scaffolds, where the pore size is determined by the spheres’ diameter, the same microgel volume in the shape of a rod has the potential to lead to much larger pores. The microgel rods are fabricated by plug‐flow microfluidics and photoinitiated free‐radical polymerization of 8‐arm star‐PEG‐acrylate (sPEG‐AC). The microgels are functionalized with either reactive epoxy groups or primary amines during polymerization on‐chip by the addition of glycidyl methacrylate (GMA) or 2‐aminoethyl methacrylate (AMA) comonomers, respectively. To interlink the two complementary microgel building blocks, the amine–epoxy addition reaction is carried out by mixing under aqueous conditions without any other required reagents. The amount of AMA significantly affects the intermicrogel assembly and interlinking efficiency, while it also influences the microgel swelling behavior, stiffness, cell adherence properties of the amine‐functionalized microgel rods. Macroscopic assemblies are achieved with pore sizes ranging predominantly from 30 to above 150 µm. When cells are seeded on the 3D microgel scaffolds, they attach, spread, and grow inside the porous generated structure.

## 2 Results

### 2.1 Microgel Rod Production via Continuous Plug‐Flow On‐Chip Gelation

Primary amine and epoxy microgel rods are produced via continuous on‐chip gelation in microfluidics. The free‐radical polymerization (FRP) crosslinking reaction is triggered by controlled UV‐irradiation inside the straight section of the microfluidic channel before the outlet utilizing lithium phenyl‐2,4,6‐trimethylbenzoylphosphinate (LAP) as photoinitiator. To apply UV‐radiation (*λ* = 365 nm) dose appropriate for crosslinking on‐chip, different irradiance settings ranging from 128 to 957 mW cm^−2^ are tested with an irradiation time of ≈2.3 s. This revealed that for higher initial AMA concentrations, a higher light intensity is needed to crosslink the microgels sufficiently on‐chip to avoid deformation after leaving the device. If 957 mW cm^−2^ is applied, all microgels retain their aspect ratio and no further flow changes are observed at higher irradiation power (Figure S1, Supporting Information).

**Figure 1:**
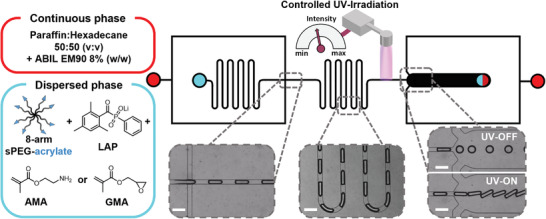
Microfluidic chip design and setup of continuous production of functionalized microgel rods via photoinitiated on‐chip gelation in plug‐flow regime. Red indicates the inlets of the first and second continuous oil phases, and cyan represents the inlet of the dispersed aqueous phase. The output is marked half red, half cyan. Dashed inserts show bright field images of characteristic sections taken during operation. Scale bars represent 250 µm.

To enable continuous collection of all types of rod‐shaped microgels, the transport of the product out of the outlet required a second flow‐focusing oil‐stream (**Figure** **1**). This oil stream avoids potential jamming of the anisometric microgels at the outlet chamber caused by the higher aspect ratio compared to spherical microgels. The chip design facilitates flushing out the crosslinked microgels at the microfluidic outlet, as the resistance is reduced (Figure S1, Supporting Information). The production rate of microgel rods employed in this study is ≈11 300 microgels per hour.

**Figure 2:**
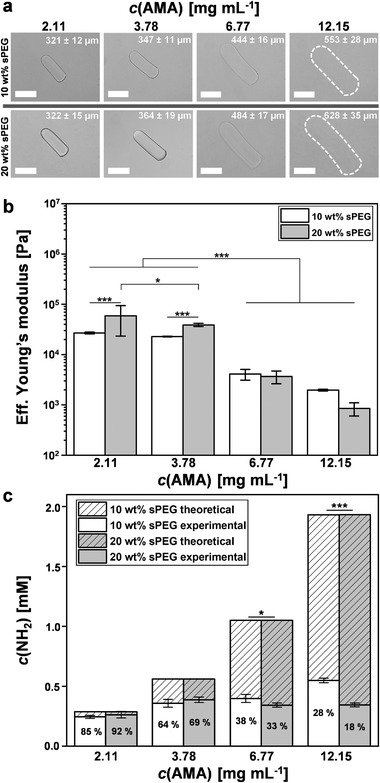
AMA concentration dependent characteristics. a) Bright field images of AMA microgel rods after purification in water with different AMA concentrations. Associated average lengths and standard deviations are indicated in each image. Data presented as mean ± SEM, *n* = 20. Dashed outlines indicate microgel rod edges. Scale bars represent 200 µm. The data distribution of all measured AMA microgel rod lengths in water and cell culture within the 10 and 20 wt% samples is shown in Figure S3 of the Supporting Information. b) Young's moduli of AMA microgel rods in PBS buffer solution (phosphate‐buffered saline: 1×, pH 7.4). Error bars represent ± SEM, *n* = 3 microgels per type at a minimum of ten different locations, *P*‐values are calculated using one‐way ANOVA with Bonferroni correction, *P < 0.05, *** *P* < 0.001. c) Theoretical and measured amine incorporation quantification of AMA microgel rods via ninhydrin assay after microgel purification. The AMA incorporation efficiency (%) is indicated within columns. Data presented as mean ± SEM, *n* = 3 per microgel type, *P*‐values are calculated using one‐way ANOVA with Bonferroni correction, *P < 0.05, *** *P* < 0.001.

During the production of all amine microgel rods, the flow rates are adjusted to maintain a microgel rod length as equal as possible (≈304 ± 14 µm; Figure S2, Supporting Information). Comparing the dimensions of swollen microgel rods from 10 to 20 wt% sPEG‐AC prepolymers with different initial AMA concentrations in water, a higher AMA concentration significantly increases the swelling of the microgel network, while the sPEG‐AC concentration does not seem to affect the swelling behavior much (Figure S3, Supporting Information). This observation could be explained by the increasing density of hydrophilic primary amines inside the microgel network, causing the structure to expand to a higher degree. In the following, we refer simply to the “AMA concentration” to distinguish between the amine‐functionalized samples based on the actual initial AMA concentration employed. In addition, a higher irradiation dose is required to crosslink microgel rods with higher AMA concentrations, suggesting a reduction in the sPEG‐AC gelation kinetics. At low AMA concentrations, the microgels slightly stiffen when the PEG concentration is doubled, which is contrasting to the observations at the highest AMA concentrations (**Figure** **2**a,b). The measured stiffness of the microgels via nanoindentation correlates well with the obtained swelling dimensions, which emphasizes the inverse correlation between the crosslinking density and the swelling degree (Figure 2b).

To quantify the primary amines within the microgel networks, a modified ninhydrin assay is employed, demonstrating that the total incorporation of the AMA comonomer increases with increase of AMA concentration for the 10 wt% sPEG‐AC microgel rods, while the average percentage of the incorporated AMA comonomer against the theoretical full incorporation decreases from 85% to 28% (Figure 2c). For the 20 wt% sPEG‐AC microgels, the total amount of copolymerized AMA reaches its highest value at 3.78 mg mL^−1^ AMA, with a slight decrease at higher AMA concentrations. Overall, AMA incorporation into the network seems to be limited up to a certain concentration and is more efficient for 10 wt% sPEG‐AC, compared to 20 wt%, at higher AMA concentrations, likely due to the reduction in solubility. As the swelling behavior of the microgels varies significantly more in contrast to the detected primary amines for different AMA concentrations, the reason for the variable mechanical properties of the microgel rods seems to also be caused by differences in on‐chip gelation, beyond the repulsive forces of the amines.

### 2.2 Microgel Assembly and Interlinking Studies

**Figure 3:**
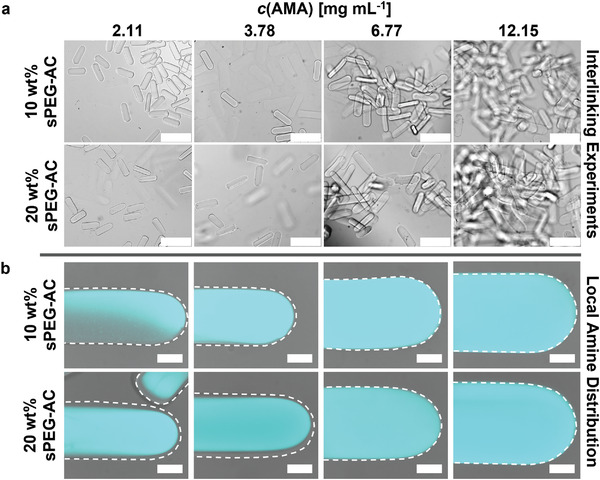
Interlinking properties between amine‐ and epoxy‐functionalized microgel rods. a) Epoxy‐functionalized microgel rods are mixed with microgel rods, synthesized with different AMA concentrations. In each case, the number of amine‐ and epoxy‐functionalized microgel rods is set to the same value. Scale bars represent 500 µm. b) Confocal laser scanning microscopy images of fluorescent FITC‐signal taken at the middle height of each microgel rod superimposed with corresponding bright field images indicating the differing amine distribution depending on AMA concentration after purification in water (cyan FITC‐signal depicts primary amine distribution). Dashed outlines indicate microgel rod edges. Scale bars represent 50 µm.

The different amine‐ and epoxy‐functionalized microgel rods are mixed to analyze their assembly behavior and interlinking efficiency into larger macroporous constructs. Successful interlinking is observed when using amine‐microgel rods with the two highest AMA concentrations. The microgel rods with lower AMA concentrations (2.11 and 3.78 mg mL^−1^) are unable to interlink efficiently with their epoxy counterparts (**Figure** **3**a). Visualization of the local amine distribution within the microgels by fluorescent covalent labeling with fluorescein isothiocyanate (FITC) revealed that the distribution strongly depends on the co‐monomer concentration (Figure 3b). At the two lower AMA concentrations for both the 10 and 20 wt% samples, an amine‐poor passivating non‐fluorescent PEG layer is observed at the outer side of the microgel, likely inhibiting interlinking with the epoxy‐functionalized microgels. Furthermore, lower AMA concentrations lead to increasingly inhomogeneous comonomer distributions within the internal microgel structure (Figure S4, Supporting Information). While it is observed that at lower AMA concentrations, the comonomer is more located in the inner part of the microgel rod, at higher AMA concentrations, amines are present throughout the entire microgel and on the surface, which is required for intermicrogel linking.

**Figure 4:**
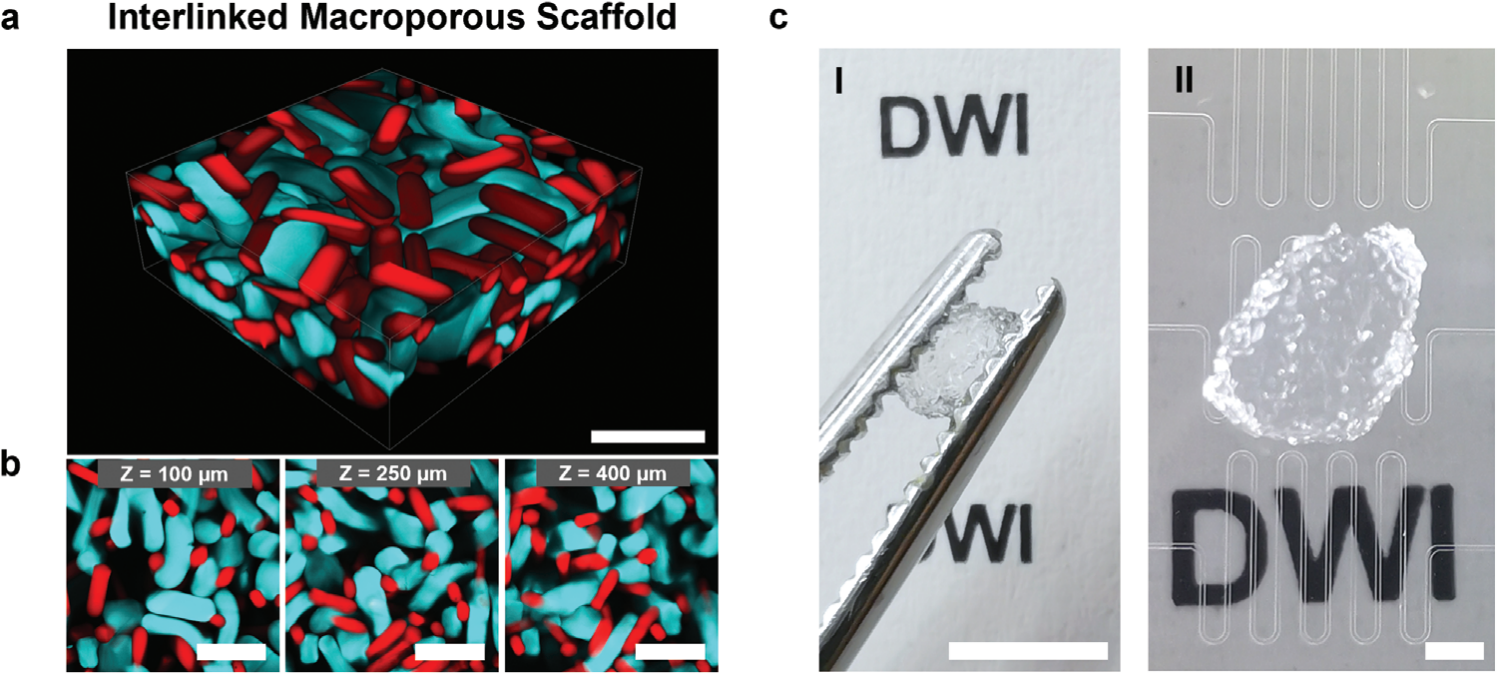
Interlinked microgel rod‐based scaffold. a) 3D projection of the 500 µm confocal microscopy Z‐stack of the interlinked scaffold made from epoxy‐functionalized microgel rods (7.90 mg mL^−1^ GMA, 10 wt% sPEG‐AC, red methacryloxyethyl thiocarbamoyl rhodamine‐B) mixed with the same number of amine‐functionalized microgel rods (12.15 mg mL^−1^ AMA, 10 wt% sPEG‐AC, cyan FITC). Scale bar represents 500 µm. b) Confocal Z‐stack images at different Z‐values (indicated in the insertion) representing the porous structure within the interlinked scaffold. Scale bars represent 500 µm. Movie S1 of the Supporting Information shows the Z‐stack. c) Images of an interlinked microgel rod‐based scaffold, between tweezers (I) and on top the employed microfluidic chips (II). Scale bars represent 5 mm in I and 1 mm in II.

The most effective interlinking is observed using amine‐microgel rods with the highest AMA concentration (12.15 mg mL^−1^). Both the 10 wt% as well as the 20 wt% sPEG‐AC microgel rods produced with the highest initial AMA‐comonomer concentration allowed for the formation of interlinked 3D structures with total volumes of ≈18 mm^3^ when assembling ≈1200 rods of each type (**Figure** **4**). The scaffold sizes currently produced in this study have a height of ≈1 to 4 mm in length and 0.5 to 2.5 mm in thickness, depending on the number of interlinked microgel rods.

In any case of interlinking 10 or 20 wt% sPEG‐AC microgel rods with the highest AMA concentration with the same number of epoxy‐microgel rods, the obtained scaffolds show a strictly alternating arrangement of the two species. If not in contact with complementary microgel species, the microgels are not built into the scaffold structure (Movie S1, Supporting Information). The interlinked scaffold can easily be transferred into another container while maintaining its stability. The soft elastic microgel network can withstand deformation caused by strong shaking or physical stress (Movie S2, Supporting Information).

**Figure 5:**
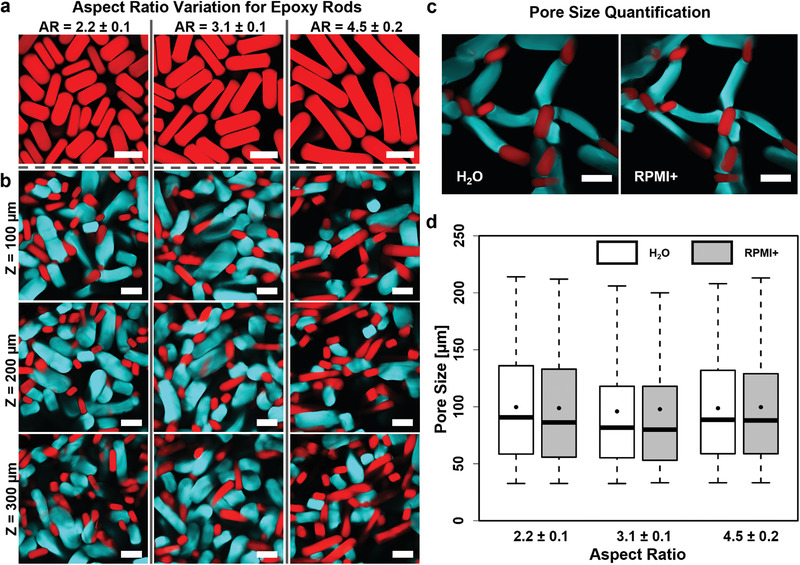
Porosity characteristics of macroporous scaffolds made via the assembly and interlinking of microgel rods with differing aspect ratios. a) Confocal images of epoxy microgel rods with differing aspect ratios (red fluorescent signal originates from copolymerized methacryloxyethyl thiocarbamoyl rhodamine‐B). Note that all microgel rods have three different dimension parameters: length, width, and height. The difference between height and width derives from the rectangular‐shaped profile of the microfluidic channel (width = 80 µm; height = 105 µm). The highest cross‐section distance value is used for the calculation of the aspect ratio. b) Confocal Z‐stack images of interlinked scaffolds from amine‐functionalized microgel rods with the AMA concentration of 12.15 mg mL^−1^ from 10 wt% sPEG‐AC in combination with epoxy‐functionalized microgel rods with different aspect ratios. Images are taken at different Z‐values (100 µm intervals). In each case, the number of amine and epoxy microgel rods is set to the same value during scaffold formation (cyan FITC‐signal depicts amine‐functionalized microgel rods; red signal visualizes epoxy‐functionalized microgel rods via copolymerized methacryloxyethyl thiocarbamoyl rhodamine‐B). c) Microgel swelling characteristics imaged at the edge of the interlinked scaffold in water (left) and RPMI+ cell media (right). Both types of microgels collapse in cell media without disrupting the interlinked scaffold. All scale bars represent 250 µm. d) Quantification of Z‐stacks of the above‐mentioned scaffolds in water and medium. The acquired data are displayed as a box plot, with the box extending from the 25th to 75th percentile and the whiskers reach out from 5% to 95% quantiles. The lines inside the boxes represent the medians, while the black points indicate means. *n* = 3 for each scaffold type. Statistical interpretation is discussed in the Experimental Section.

To analyze the effect of the microgel aspect ratio on the macroporosity of the assembled scaffold, epoxy‐functionalized microgel rods are produced with 10 wt% sPEG‐AC and different aspect ratios (2.2 ± 0.1; 3.1 ± 0.1, and 4.5 ± 0.2) (**Figure** **5**a) and combined with amine‐functionalized microgel rods (10 wt% sPEG‐AC, aspect ratio: ≈2.9 ± 0.2, 12.15 mg mL^−1^ AMA). Using the same number of complementary rods (≈1200) in every interlinking experiment, all three aspect ratios of the epoxy microgels result in stable interlinked scaffolds. 90% of the pore sizes for all samples range from 30 to above 150 µm, while the mean values are near 100 µm (Figure 4d).

In addition, sphere‐like microgels (aspect ratios of 1.16 ± 0.06 with length of 143 ± 8 µm and diameter of 123 ± 4 µm for amine‐functionalized microgels, and 1.42 ± 0.03 with length of 154 ± 4 µm and diameter of 109 ± 3 µm for epoxy‐functionalized microgels; *n* = 50) are produced on‐chip, irradiated at the same spot at similar flow rate, resulting in the same UV‐dose as for the microgel rods. They are also interlinked into scaffolds via amine–epoxy chemistry as a reference model. Scaffolds formed from sphere‐like microgels are unstable as the microgels interlink before they can densely pack. This results in wider pore size distributions with some regions of the construct densely packed, while others have a small number of microgels that do not efficiently bridge the structure throughout the volume, negatively impacting the stability of the scaffold (Figure S6a,b, Supporting Information). This irregular density profile may be influenced by the employed chemistry as other reports successfully produced stable MAP scaffolds from spherical microgels.^[^ ^5^, ^6^, ^17^ ^]^ The microgel interlinking in our report is achieved by bringing two complementary microgel components into contact with each other without any further additives and takes around 2 to 3 s. This leads to an inhomogeneous sphere‐like microgel assembly that encounters a nonhomogeneous force distribution when the system is exposed to mechanical stress, resulting in fragmentation into smaller parts. Larger fragments of dense interlinked sphere‐like microgel assemblies are analyzed similar to the assemblies of the rod‐shaped microgels with respect to pore size distribution and porosity (Figure S6d,e, Supporting Information). There, pore sizes are observed between ≈10 and 55 µm with a mean value around 22 µm, which is in accordance range with the reported publication by Segura group.^[^ ^5^, ^6^ ^]^ The dense construct fragments exhibit an overall porosity between 20% and 50%. In the case of microgel rods, stable constructs are formed as the rods are able to bridge the overall construct volume, resulting in 4.5‐fold larger pores with less overall microgel volume (overall porosity between 40% and 70%, Figure S8, Supporting Information) in comparison to the sphere‐like assemblies.

The comparison between sphere‐like and rod‐shaped microgels confirms that larger pores can be obtained in a stable scaffold using less material per unit volume with rod‐based microgel systems. Additionally, the volume ratio of the two different microgel types inside the scaffold can be varied while keeping the number of rods equal by varying their aspect ratio. This has the potential to alter the biochemical functionality of the scaffolds without significantly changing their porosity or stiffness. Modification of the microgels can be performed on‐chip or postproduction with the active amine and epoxy‐functional groups remaining after interlinking into the macroporous scaffold.

### 2.3 L929 Cell Studies on Individual Microgel Rods

**Figure 6:**
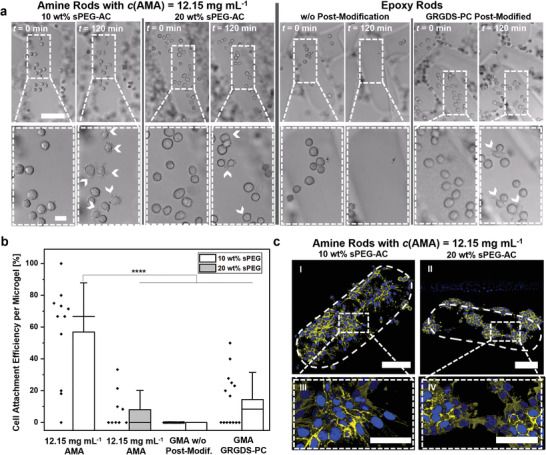
L929 fibroblast adhesion on individual microgels. a) Bright field images showing cell attachment during live imaging at *t* = 0 and 120 min. White arrowheads indicate filopodia‐like protrusion involved in cell–material interaction. Amine‐functionalized microgels modified with GRGDS‐PC (on‐chip production) show filopodia protrusions at *t* = 120 s for 10 wt% PEG‐AC, while cell attachment observed for 20 wt% PEG‐AC shows more rounded cells. Epoxy‐functionalized microgels exclusively show cell attachment after postmodification with GRGDS‐PC. The scale bar in the top and bottom row represents 125 and 20 µm, respectively. b) Quantification of L929 attachment efficiency on different microgel surfaces is performed after a time period of *t* = 120 min. Cell attachment efficiency per microgel is computed by counting the fraction of initial cells on the microgel surface (*t* = 0 min) that show substrate interaction, marked by filopodia protrusions, within this time period. Data presented as mean ± SD. Data points are displayed as black rhombuses adjacent to the bars (minimum‐*n* = 9 microgel rods). *P*‐values are calculated using one‐way ANOVA with Bonferroni correction, **** *P* < 0.0001. c) Fluorescent confocal images display cell spreading and growth on individual microgel rods after 4 days of culture. Microgel outlines are indicated with white dashes. The scale bars in images I and II represent 100 µm while the scale bars in III and IV represent 50 µm. The cells are stained for actin (yellow) and the cell nuclei (blue).

L929 mouse fibroblast cells are seeded on the surface of individual amine‐ and epoxy‐functionalized microgel rods, capable of producing interlinked scaffolds, to analyze cell–microgel interaction. Amine‐functionalized microgels are modified with GRGDS‐PC during on‐chip production, introducing distinct cell adhesion sequences within the microgels. The peptide contains a cysteine, which can couple to free acrylates via thiol‐Michael addition. Epoxy‐functionalized microgel rods are fabricated with and without GRGDS‐PC modification. Here, GRGDS‐PC is incorporated in the microgels after they are interlinked via the free amine of the glycine and the remaining epoxy groups to avoid quenching of epoxy‐functionalities during microgel production. As expected, cells exclusively interacted with GRGDS‐modified epoxy microgel rods. Cell adhesion on the microgels is monitored during 120 min at 37 °C, 5% CO_2_ in humid environments. These live observations reveal that cell–substrate interactions are more distinctive for higher amine‐concentration (**Figure** **6**a; Figure S7a, Supporting Information). Additionally, cell attachment is more prominent on the microgels fabricated with a sPEG‐AC concentration of 10 wt% compared to 20 wt%. This is surprising as fibroblasts normally spread better on stiffer elastic hydrogel surfaces and may be related to a higher concentration of positively charged amine‐groups on the microgel surface. For 10 wt% sPEG microgels with the highest AMA‐concentration, ≈70% cell attachment efficiency per microgel is observed (Figure 6b). All other amine‐conditions show significantly lower cell attachment efficiencies, as most cells slide off the microgel surface or show no distinct substrate interaction.

After 4 days of culture, the cells are fixed, and their nucleus and actin filaments fluorescently labeled to analyze cell spreading. When comparing 10 and 20 wt% microgel rods with different AMA concentrations, the 10 wt% sPEG microgels with the highest AMA concentration demonstrated the highest number of spread cells (Figure 6cI,III). During culture, the fibroblasts proliferated and almost covered on the entire microgel surface. Meanwhile, for 20 wt% sPEG microgels, the cells formed cluster‐like assemblies on the microgel surface, suggesting reduced cell‐microgel interaction (Figure 6cII,IV). Similar trend is observed for lower AMA‐concentrations (Figure S7c, Supporting Information).

### 2.4 L929 Cell Culture inside Interlinked Macroporous 3D Scaffolds

**Figure 7:**
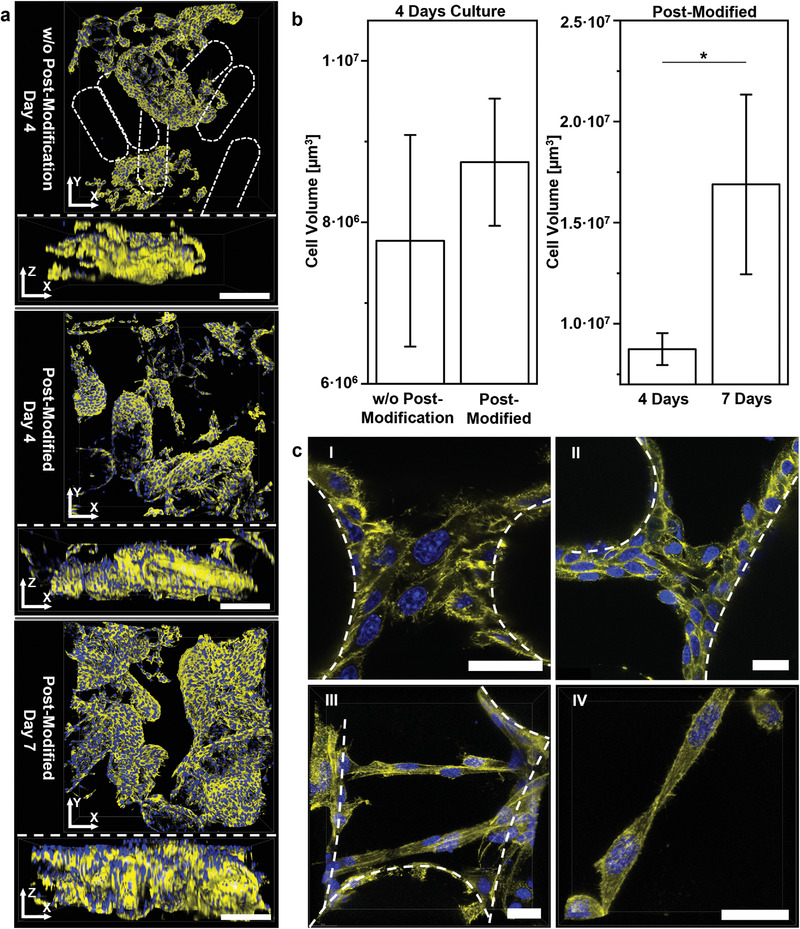
3D fibroblast growth inside interlinked macroporous microgel scaffolds. a) 3D representations of 250 µm confocal Z‐stacks of L929 cells after 4 and 7 days culture inside macroporous scaffolds (above *XY*‐view; below *XZ*‐view). Scale bars represent 200 µm. Amine‐functionalized microgels are modified with GRGDS‐PC during on‐chip crosslinking, epoxy‐functionalized microgels can be postmodified with GRGDS‐PC to not interfere with the epoxy groups during interlinking. From top to bottom: Without postmodification of the epoxy‐functionalized microgels, the cell network resulted in less continuous cell structures as cells only attach to the amine‐functionalized microgels with GRGDS‐PC (microgel rods without attached cells are indicated via dashed outlines). More interconnected cell structures are observed for scaffolds using epoxy microgel rods postmodified with GRGDS‐PC with increasing cell growth between 4 and 7 days culture. b) Cell volume quantification for Z‐stacks comparing with and without postmodification of epoxy‐functionalized microgels with GRGDS‐PC and cell growth over time in the case of postmodification. Note that the postmodified data after 4 days culture is used in both graphs. Data presented as mean ± SEM, *n* = 3. *P*‐values are calculated using one‐way ANOVA with Bonferroni correction, *P < 0.05. c) Bridging cell structures at day 7 of culture inside scaffolds, postmodified with GRGDS‐PC, with dashed outlines indicating microgel rods. All scale bars represent 25 µm. I and II: Confocal microscopy overlay scans displaying cell structures covering and bridging neighboring microgel rods. III: Superimposed confocal microscopy 50 µm Z‐stack showing bridging cell structures formed in a large pore at different heights. IV: Superimposed confocal 25 µm Z‐stack of a bridging structure formed by two cells. For all images: yellow phalloidin‐iFluor 594‐signal depicts actin; blue DAPI‐signal visualizes cell nuclei.

When seeding approximately equal numbers of L929 cells on macroporous microgel scaffolds with and without postmodification with GRGDS‐PC, both structures result in cell spreading and growth along the microgel surface after 4 and 7 days in culture (**Figure** **7**a,b). In the case of unmodified epoxy microgels, the observed cell structures are less interconnected compared to the postmodified samples (Figure 7a). The epoxy‐functionalized rods without RGD show less interaction with cells and prevent the formation of an interconnected cell growth throughout the 3D scaffold. Investigating the cell structures formed within scaffold pores, different bridging structures at higher or lower cell densities are observed (Figure 7c). As the cells inside the macroporous microgel assemblies do not need to degrade the hydrogel to grow in 3D, their cytoskeleton can spread out reaching for the neighboring rods. To further investigate the cell–microgel interaction and characterize cellular tissue formation, fibronectin, a natural protein produced by the cells is stained after 7 days of L929 cell culture within interlinked microgel rod‐based scaffolds. Fibronectin is localized between the cells in the macropores and at the interface between the microgels and cells, showing a clear interaction with the scaffold.^[^ ^18^ ^]^ Overall, a large variety of cell structures at different angles and cell densities is determined in every interlinked scaffolds assembled with microgel rods.

### 2.5 Human Fibroblast and Endothelial Cell Culture Studies

To study the suitability of the same type of microgel rod scaffolds for additional cell types, human fibroblasts are seeded with approximately the same cell number on individual amine‐functionalized microgel rods and the already described microgel rod scaffolds. After different time periods in culture a mixture of HUVECs and human fibroblasts (ratio 1:3) is seeded as a subsequent step. The cell number ratio is chosen because of prior studies in our group showing best HUVEC proliferation results in 3D bulk hydrogel experiments. Based on the previous L929 model, human fibroblasts and HUVECs are added after 5 and 7 days of the exclusive human fibroblasts culture. The idea is to achieve a layer of human fibroblasts on the surface of the microgel scaffold and, in a second step, use the formed macroporous network for HUVEC proliferation aiming for vessel structure formation.

**Figure 8:**
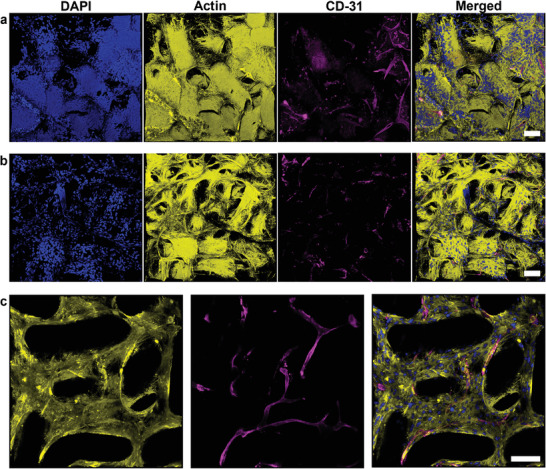
Confocal imaging of human fibroblasts and HUVECs after culture inside macroporous 3D scaffolds. a) Human fibroblasts cultured inside an interlinked microgel rod scaffold for 5 days before HUVECs and additional fibroblasts (ratio 1:3) are added and cultured for 14 more days. b) Human fibroblasts cultured inside a microgel rod scaffold for 7 days before HUVECs and additional fibroblasts (ratio 1:3) are added and cultured for 16 more days. c) Z‐stack taken near the edge of the same scaffold imaged in (a) to (c) is superimposed 3D representations of 150 µm Z‐stacks for (a) and (b), and 100 µm Z‐stack for (c). (For all images: yellow phalloidin‐iFluor 594‐signal depicts actin; blue DAPI‐signal visualizes cell nuclei; magenta shows CD‐31/PECAM‐1 labeled HUVECs; all scale bars represent 100 µm.)

Interestingly, human fibroblast proliferation turns out to be more efficient compared to L929 fibroblasts. Human fibroblasts are able to attach, spread, and grow on the microgel rods and in the case of the 3D scaffolds even fill most of the pores completely after 5 days (**Figure** **8**a). With further culture time, cells continue to fill up the macropores. This observation hints towards sufficient nutrient supply from cell culture media to the cells within the scaffold, which is supported by diffusion through the interconnected permeable microgel network. As the pores are not much larger than 200 µm, necrotic tissue is not likely to be formed inside the macropores, also not in the center of the scaffold. In the current situation, the rod‐shaped microgels function as artificial “blood vessels” to bring oxygen and nutrients to the cells. When the microgels would degrade over longer periods of time and be replaced by tissue, diffusion via the microgel network will be impaired and necrosis will occur if there is no vascular network. For this reason, first experiments are performed toward creating blood vessels in these macroporous constructs via vascular cocultures. Here, sequential seeding of fibroblasts and a fibroblast‐HUVECs mixture at different time points was performed, resulting in platelet endothelial cell adhesion molecule (PECAM‐1) signals in between the microgels, as well as on the microgel surface, demonstrating endothelial cell growth and spreading. However, HUVEC‐cell spreading and growth along with the first stages of intercellular assembly into elongated superstructures is observed (Figure 8a,b). Even more developed elongated structures can be found toward the edge of the microgel rod scaffold (Figure 8c), which is most likely due to the more open structure toward the edge of the scaffold, compared to the pores in the center of the scaffold that are almost completely filled by fibroblasts (Figure S9b, Supporting Information). To systematically analyze human fibroblast‐ and HUVEC‐proliferation inside our macroporous 3D scaffolds, these findings are used in the upcoming studies to design a more extensive series of experiments.

## 3 Discussion

To our knowledge, this report is the first one to assemble and interlink a two‐component system of synthetic rod‐shaped microgels with defined dimensions, stiffness, and reactivity into a 3D macroporous hydrogel scaffold. These constructs provide an open and cell‐supportive environment with variable architecture, biochemical cues, and mechanical properties to create 3D cell networks. The main goal of this study is to replace spherical microgels with soft anisometric building blocks to achieve larger pores with less material in randomly assembled structures for tissue engineering. The microgels are produced by free‐radical polymerization of sPEG‐AC and reactive comonomers using droplet generator microfluidics, while the microgel rod length can be set to a preferred value allowing for the variation of the aspect ratio. To achieve sufficient crosslinking on‐chip and allow for efficient and stable continuous collection of the microgel rods, the chip design is combined with a custom‐made controlled UV‐irradiation light source and optimized with introducing a second flow‐focusing oil. These adjustments are essential to preserve the anisometric geometry compared to spherical microgels. If the microgel rod length and the crosslinking ratio increase, formation of a flow vortex inside the outlet becomes more probable caused by an increasingly complex flow interference between the microgel rods, which could impede controlled operation. The second flow‐focusing oil phase avoids excessive jamming of the microgel rods inside the outlet, which would lead to periodic pressure changes in the channel, negatively affecting the geometry of the produced microgel rods.

The aim of this study is to interlink complementary amine‐ and epoxy‐functionalized rods into 3D macroporous constructs. Our results demonstrate that with increasing AMA concentration, the absolute number of primary amines only marginally increases and thus the incorporation efficiency of the reactive group decreases (Figure 2c). In the case of 20 wt% sPEG‐AC microgels, the measured amount of primary amines even starts to stagnate when the initial AMA concentration reaches 6.77 mg mL^−1^. The higher swelling behavior and lower stiffness (Figure 2a,b) indicate that an increasing AMA concentration reduces the crosslinking density of the microgel rods and results in a more homogeneous amine distribution throughout the microgels. Moreover, a higher PEG weight percentage (wt%) unexpectedly results in softer and more swollen microgels in the case of AMA concentrations of 6.77 and 12.15 mg mL^−1^, compared to the samples with lower PEG weight percentages or lower AMA concentrations (Figure 2a,b). The incorporation and distribution of amine groups throughout the microgel network and the crosslinking density (stiffness, swelling of microgel) appear to be influenced by the initial AMA concentration but also by the ratio of AMA to sPEG‐AC. The local distribution of primary amines changes from being mainly within the core of the microgels for the two lower AMA concentrations to the entire microgel network, including the surface, for the higher AMA concentrations. The presence of reactive amines on the surface of the microgels is required for microgel interlinking with the complementary epoxy‐functionalized rods, which is indeed successful at the higher AMA concentrations (Figure 3b). The density of active functional groups for interlinking and their accessibility are crucial to create stable 3D constructs.

As the 10 wt% sPEG‐AC‐based microgel rods resulted in a better AMA incorporation efficiency for an AMA concentration of 12.15 mg mL^−1^, they were selected to interlink with the epoxy‐functionalized microgels. Mixing the same number of amine‐ and epoxy‐functionalized microgels with comparable aspect ratios results in interlinked scaffolds within 2–3 s without further additives, where all the rods are consumed to form macroporous flexible and yet stable alternating structures (Figure 4c; Movies S1 and S2, Supporting Information). The obtained dimensions of pore sizes are predominantly in the range from 30 to above 150 µm (Figure 5). Variation of the aspect ratio of the epoxy‐functionalized microgels from 2.2 to 4.5 did not significantly change the overall pore‐size distributions of the constructs, likely due to the fact that the microgels are reactive over their entire surface area. This demonstrates that the amount of biochemical signals provided by each kind of microgels could be altered without drastically changing the open structure. The comparison of the macroporous size distributions and the resulting stability properties of rod‐shaped and sphere‐like microgels demonstrate that larger pores can be obtained in a stable scaffold using less material per unit volume using rod‐based microgel systems (Figure S6, Supporting Information).

Microgels are modified with the GRGDS‐PC peptide to render them cell‐adhesive. While the amine‐functionalized microgels can be modified on‐chip, the epoxy‐microgels are postmodified after assembly and interlinking the 3D construct to prevent interference with the reactive epoxy groups. As expected, unmodified epoxy microgel rods do not demonstrate efficient cell adhesion, while postmodified microgels with the cell‐adhesive peptide support cell attachment, spreading, and growth (Figure 6). Interestingly, the microgels made with higher AMA concentrations lead to more efficient cell attachment and growth. This is surprising as elastic synthetic hydrogels usually support cell spreading on stiffer hydrogels with Young's moduli above ≈3 kPa.^[^ ^19^ ^]^ Even for MAPs made from spherical microgels, an increased microgel stiffness (3.6 kPa vs 1.6 kPa) led to enhanced cell spreading and better cell growth.^[^ ^6a^ ^]^ The enhanced cell growth on our softer microgels may be due to an increased concentration of positively charged amines on the surface of the microgels. Previous reports on primary amines have shown positive effects on stem cell viability, as well as neuronal differentiation and neurite extension.^[^ ^20^ ^]^ Classification of the influence of gel stiffness against the influence of the chemically active surface of the microgels is under further investigation and is beyond the scope of this study.^[^ ^21^ ^]^

In combination with live imaging analysis to probe L929‐cell attachment and spreading on individual GRGDS‐PC‐modified microgel rods, confocal microscopy of fixed and stained cultures demonstrated that the interlinked scaffolds feature strong cell adhesion and support cell growth along the microgel surface in culture. The same trend is successfully reproduced in the macroporous 3D cultures (Figure 7; Figure S9, Supporting Information). The GRGDS‐PC postmodified scaffolds show more interconnected cell spreading throughout the scaffold volume. If the remaining epoxy groups are not postmodified, cells favor the amine‐functionalized microgels to grow on the surface of the macroporous scaffold. Cells proliferate and migrate in the assembled microgel constructs and fill the scaffold pores over the duration of culture, while the cells have the ability to form structures bridging multiple microgels across the pores (Figure 7c). Additional fibronectin immunostaining shows that cells produce fibronectin that is localized between the cells and in the macropores, and is present at the interface between the microgels and cells, showing a clear interaction with the scaffold and the onset of tissue formation (Figure S10, Supporting Information). Native fibronectin production supports endothelial cell sprouting and blood vessel formation.^[^ ^22^ ^]^ As native fibronectin is observed after cell culture within interlinked microgel rod‐based scaffolds at the interface between the microgel rods and cells and also in between the cells present in the macropores, it is expected to promote tissue vascularization by endothelial cells in the future.

The bottom‐up fabrication method and its versatility enables postmodification via active epoxy groups with different, and potentially more specific, cell‐responsive moieties than GRGDS‐PC using other thiol or primary amine compounds. Human fibroblasts show an even more efficient cell growth. After 5 to 7 days in culture, human fibroblasts fill most of the macropores resulting in a stable tissue‐like structure that withstands mechanical stress during sample manipulation. At that time, an additional coculture of HUVECs and human fibroblasts are seeded in a 1 to 3 ratio. After 14 to 16 more days in culture, HUVECs spread and grow inside the scaffold resulting in the first elongated intercellular structures necessary towards potential vessel formation. As human fibroblast proliferation turned out to be more efficient than expected, the upcoming experiments will analyze the dynamics of pore occupation by human fibroblasts aiming for the optimal conditions for HUVEC‐cell addition to allow for vessel formation. As larger macropores and higher total porosity can be achieved with microgel rod‐based constructs and cells grow and fill the pores over time, the resulting volume ratio of cells to scaffold is much higher compared to spherical‐based microgel constructs. The higher number of cells inside the macropores enhances cell–cell communication, which is important for many physiological processes. This level of control would allow to grow different cell types in a more spatially compartmentalized manner, which could also be implemented in bioprinting technologies to grow organized and functional 3D tissues. Further versatility of pore geometries can be realized by changing the aspect ratio and dimensions of the microgels to a larger extent. In the future, the quick interlinking between the amine‐ and epoxy‐functionalized microgels could be utilized for creating more complex construct geometries by controlling the mixing of the two components on the micro‐ and millimeter‐scale during extrusion.

## 4 Conclusion

We believe that these synthetic building blocks with variable properties and the resulting architectures made via a bottom‐up mechanism hold high potential for customized soft bio‐interactive scaffolds for 3D cell culture and tissue growth. The novel 3D macroporous constructs described here could form the basis for a new biomaterial standard to grow 3D in vitro tissues to enable drug and therapy testing. The diverse nature of the pore geometry and microgel properties will enable proprioceptive studies to investigate cell behavior in terms of their orientation relative to the microgel surfaces and pores in conjunction with specific cell–material and improved cell–cell interactions. In particular, they could be beneficial for blood vessel ingrowth into the growing tissue to provide nutrients and oxygen. Artificial vascularized tissues are crucial for disease modeling and environmental effect studies.

## 5 Experimental Section

### Chemicals, Cells Lines, and Cell Culture Material

The following materials were purchased and used as per the instruction. ABIL EM 90 (Evonik Nutrition), 2‐aminoethyl methacrylate hydrochloride (Sigma‐Aldrich, 90 %), anti‐fibronectin antibody, rabbit polyclonal to fibronectin (abcam), 8‐arm PEG‐acrylate 20 kDa (Biochempeg Scientific Inc., ≥95%), 4′,6‐diamidino‐2‐phenylindole (Thermo Fisher Scientific), Dulbecco's modified Eagle medium (DMEM, Gibco), endothelial cell growth medium (EGM‐2, Promocell), ethanol (VWR‐chemicals, 99.8%), fluoresceinamine isomer I (Sigma‐Aldrich), fluorescein isothiocyanate (Thermo Fisher Scientific), glycidyl methacrylate (Sigma‐Aldrich, 97%), glycin (Thermo Scientific, ≥99%, ultrapure), goat anti‐mouse secondary antibody, Alexa Fluor 633 (Invitrogen), GRGDS‐PC (H‐Gly‐Arg‐Gly‐Asp‐Ser‐Pro‐Cys‐OH (trifluoroacetate salt), CPC Scientific), hexane (Sigma‐Aldrich, >99%), human CD31/PECAM‐1 antibody (primary, monoclonal mouse, R&D systems), HUVECs (Lonza), lithium phenyl‐2,4,6‐trimethylbenzoylphosphinate (Sigma‐Aldrich, ≥95%), methacryloxyethyl thiocarbamoyl rhodamine‐B (Polyscience), ninhydrin reagent 2% sol. (Sigma‐Aldrich), normal human dermal fibroblasts (Promocell), Novec 7100 (Sigma‐Aldrich), oil red o (Sigma‐Aldrich), paraffin (VWR‐chemicals), paraformaldehyde (Sigma‐Aldirch), phosphate buffered saline (PBS, pH = 7.4, *c* = 1×, Thermo Fisher Scientific), phalloidin‐iFluor 594 reagent (abcam), 2‐propanol (99.5%), RPMI 1640 medium (Gibco), SYLGARD 184 silicone elastomer kit (Dowsil), and toluene (VWR‐chemicals, 99.5%).

### Preparation of Prepolymer Solutions

All prepolymer solutions were stored in brown glass vials to prevent undesired photoinitiator decomposition. Primary amine‐functionalized microgels were prepared from 8‐arm sPEG‐AC (molecular weight (Mw) = 20 kDa) and LAP photoinitiator dissolved using freshly prepared aqueous solutions of AMA hydrochloride with four different concentrations (2.11, 3.78, 6.77, 12.15 mg mL^−1^). The AMA solutions were passed through a CHROMAFIL MV A‐20/25 syringe filter before mixing. The final LAP concentration was set to 1 wt% and sPEG‐AC concentrations to either 10‐ or 20 wt% in aqueous solution. A GRGDS‐PC solution was added subsequently to the final concentration of 1 × 10^−3^ m. Epoxy‐functionalized microgels were prepared from aqueous solutions of LAP (1 wt%) used to dissolve 8‐arm sPEG‐AC (Mw = 20 kDa) to a final concentration of 10 wt% and subsequent addition of distilled GMA with a final concentration of 7.90 mg mL^−1^.

### Continuous Plug‐Flow On‐Chip Gelation Microfluidics

Microgel rods with variable aspect ratios were obtained via on‐chip gelation in a flow focusing microfluidic device with a channel diameter of 80 µm operated in plug‐flow regime. The optimized chip design comprises one inlet for the dispersed phase, two inlets for the continuous phase, and one outlet for product removal. Flow rates were controlled via Gastight syringes and PHD ULTRA syringe pumps by Harvard Apparatus. Colored microfluidic devices were produced to prevent undesired scattering of the light and kept the spot size focused as previously reported.^[^ ^14b^ ^]^ The composition of the disperse phase was characterized as reported in the previous subsection, while the continuous phase was formed by a mixture of paraffin oil and hexadecane (volume ratio = 1) with 8% (w/w) of ABIL EM90 as surfactant. On‐chip crosslinking of the dispersed phase was initiated through continuous irradiation using a self‐constructed UV‐LED (*λ* = 365 nm; spot diameter ≈ 4.7 mm) providing controllable irradiance (at 957 mW cm^−2^) in the straight part of the channel through the glass side of the microfluidic device (Figure 1). Each droplet of the disperse phase passed the irradiated area in ≈2.3 s. The aspect ratio inside the channel and the number of collected AMA‐microgel rods over time were set to the same value during on‐chip gelation (Figure S2, Supporting Information). To avoid clogging of the rods in the outlet chamber of the chip, a second flow‐focusing oil‐stream was added to guide the rods away from the outlet in a more focused and rapid manner. Purification of the obtained microgels was carried out by multiple subsequent washing with hexane, isopropanol, and water as previously reported.^[^ ^14c^ ^]^

### Mechanical Quantification via Nanoindentation

The micromechanical properties of the microgels were obtained with the high‐throughput mechanical screening platform Pavone (Optics11Life, Amsterdam, The Netherlands). Per type, three microgel rods were indented in the center at a minimum of ten different locations. Indentation measurements were performed using a cantilever‐based probe with a spherical tip radius of 10.5 µm and a cantilever stiffness of 0.25 N m^−1^. The piezospeed is 15 µm s^−1^ and the indentation‐depth is 2 µm. From the obtained load–indentation curves, the Hertzian contact model was used to calculate the effective Young´s modulus *E* (kPa).^[^ ^23^ ^]^ All measurements were performed at room temperature.

### Ninhydrin Assay to Quantify Primary Amine Incorporation

For all measurements, the volume ratio of 2% ninhydrin solution to the sample was set to the same value (0.8). The calibration was carried out from samples with defined glycine concentrations in the relevant final concentration range for the later analyzed samples. To quantify different microgel rod dispersions, on‐chip gelation for each sample was conducted until the same volume of the disperse phase was obtained. Microgel samples for ninhydrin analysis were collected in separate vials directly from microfluidics and quantified after purification. After addition of ninhydrin solution, the mixture was heated for 30 min at 80 °C, shaken, and centrifuged, after which 100 µL of the microgel supernatant were transferred into a 96‐well plate with subsequent addition of 100 µL ethanol. The resulting absorbance measurements at *λ* = 570 nm were performed in a Tecan Sunrise absorbance reader 12 min after ethanol addition (triple determination).

### Qualitative Determination of Functional Groups via Confocal Laser Scanning Microscopy (CLSM)

Confocal laser scanning microscopy was performed on a Leica TCS SP8 microscope (Leica Microsystems, Germany). In aqueous dispersions of microgel rods, covalent labeling of epoxy groups was achieved by the addition of 6‐aminofluorescein (0.5 mg mL^−1^), while amine functional groups were labeled via FITC (0.5 mg mL^−1^) in DMSO. Excess unbound fluorescent marker was removed by multiple solvent exchanges of the supernatant after sedimentation. 6‐aminofluorescein and FITC were excited at *λ* = 488 nm via an argon laser. The HC PL APO 63X/1.30 glycerol objective with glycerol immersion liquid type G (Refractive index: 1.45) was used to detect the resulting emission at *λ* = 500–550 nm.

### Assembly and Interlinking of Functionalized Microgel Rods

Interlinking experiments were performed between microgels with different amine concentration and epoxy‐functionalized microgel rods in deionized water (volume 100 to 700 µL) overnight at room temperature. The same number of the two different microgel components was used in all conditions (ranging from ≈100 to 1200 rods each). Mixing of amine and epoxy rods was ensured by manual pipetting via an Eppendorf pipette. The resulting scaffold porosity was investigated for epoxy‐functionalized microgel rods with different aspect ratios (2.2 ± 0.1, 3.1 ± 0.1, and 4.5 ± 0.2). To remove loose non‐interlinked microgel rods, the resulting structures were rinsed several times with deionized water. Formation of the complementary‐alternating interlinked microgel rod scaffolds was confirmed using confocal laser scanning microscopy as described above.

### Determination of the Spatial Distribution of Microgel Rods via CLSM in 3D

Copolymerized methacryloxyethyl thiocarbamoyl rhodamine‐B (1 vol% of a 10 mg mL^−1^ solution in DMSO) was used to label epoxy‐functionalized microgel rods in order to track their position in the interlinked scaffold. Rhodamine‐B was excited at *λ* = 561 nm using a DPSS laser and the emission was detected at *λ* = 596–650 nm. In the case of microgel rod scaffolds, the FITC labeling of residual amines (as described above) was performed as the last step after interlinking the rhodamine‐B‐stained epoxy microgels with unlabeled amine microgels to avoid affecting the interlinking of the complementary microgels with each other.

### Pore Size Distribution and Porosity

Individual scan overlays of amine‐ and epoxy‐functionalized microgel rods were collected using confocal microscopy as Z‐stacks. The microgels showed no closed pore structure but a continuous background structure. Therefore, an algorithm was built to establish the pore size by estimating the lowest local half‐width of this background in each slice. The individual images within the stacks were converted to gray scale such that both channels were normalized to their common maximum intensity value. In order to remove out of focus parts, a background image was made blurring the original through convolving it with a Gaussian kernel (window width: 301 pixels, standard deviation: 50 pixels), which background was then subtracted. Resulted negative values were set to zero. The image was then smoothened convolving it with another, this time narrow Gaussian kernel (window width: 13 pixels, standard deviation: 2 pixels). Objects were detected as the 75th percentile of the image intensity. The binary background image (object pixels are 1, the rest is 0) eroded twice and then dilated twice to remove single pixel noise. Counting the produced background pixels allows for estimating the porosity in each image slice as the ratio of background pixels/all image pixels. Because in several stacks the volume of the sample did not fill the whole image, this measure is prone to a high error, especially at the top and bottom of stacks (Figure S8, Supporting Information).

To determine the sizes in the background structure, a distance filter was used, where the ridges were identified as local maxima. The distance filter produced a quadratic cartesian distance from the closest edge.^[^ ^24^ ^]^ Searching for the local maxima employed a second derivative filter with identifying maxima higher than 12 pixels distance step (https://launchpad.net/imagep).

A simple algorithm also identified where the structures ended on the image and removed the distance estimation between the structure and the edge of the images. The resulted ridge points were collected values converted to distance with calculating their square root, and then exported. The analysis was written in python (https://www.python.org) and is available as the ImageP packaged on Launchpad (https://launchpad.net/imagep). Each stack provides several thousands of data points, up to millions of data points per sample type. This causes standard statistical methods, such as Student's *t*‐test to result in very low probabilities rendering such comparisons not applicable to this data set.

### Interaction Analysis between Cells and Microgels via Live Imaging

Aqueous microgel dispersions were UV‐sterilized for 30 min. Following, the microgels were washed three times with sterile PBS solution (1 m) and RPMI supplemented with 10% fetal bovine serum (FBS) and 1% antibiotic‐antimycotic (AMB), respectively. A droplet (40 µL) of the microgel suspension was cast on a glass‐bottom petri dish (ibidi, GmbH). L929 fibroblasts were added at a concentration of 1000 cells µL^−1^. Live images were recorded at 37 °C and 5 vol% CO_2_ on a ZEISS Axio Observer Z1 inverted microscope. Images were captured every 30 s at a fixed Z‐position for a time span of 120 min. Cell attachment efficiency per microgel was computed by counting the fraction of initial cells on the microgel surface (*t* = 0 min) that showed substrate interaction, marked by filopodia protrusions, within this time period.

### Cell Culture

The samples were cultivated at 37 °C and 5 vol% CO_2_ for 7 days inside the macroporous scaffolds, which were placed in a transparent cell culture insert with a pore diameter of 3 µm (ThinCert) after interlinking and postmodification. Media (RPMI+) was carefully exchanged through the insert membrane on the second day of cultivation. The samples were then washed once with PBS (1 m) for 5 min and fixed under the addition of 4% paraformaldehyde for 30 min at room temperature through the insert membrane, followed by washing with PBS for 15 min, 0.1% TritonX‐100 (v/v) for 3 min, and again with PBS for 15 min. F‐actin filaments were stained using phalloidin‐594 (1:1000 Abcam Phalloidin‐ifluor in PBS) for 1 h, followed by washing twice with PBS. Fibronectin signal was obtained by first adding a blocking solution (4% BSA/PBS) for 2 h followed by the addition of primary antibodies against fibronectin (diluted 1:200) over night, and then washing three times with PBS. Then the secondary antibodies (diluted 1:200) and phalloidin‐633 (diluted 1:1000) were added for 1 h and washed three times with PBS. Nuclei stains were achieved by the addition of 4′,6‐diamidino‐2‐phenylindole (DAPI) (1:100 in PBS) for 20 min followed by washing twice with PBS.

For the human cell lines, the human fibroblasts were cultivated with DMEM supplemented with 10% FBS and 1 % AMB (DMEM+) at 37 °C and 5 vol% CO_2_ inside the macroporous scaffolds in the inserts. After 5 or 7 days of culture, HUVECs and human fibroblasts are added (1:3 ratio) to the preseeded fibroblasts and cultured for another 14 or 16 days in EGM and DMEM+ media (1:1 ratio). The media was changed every two days. The samples were fixed the same manner as for mouse fibroblasts. After permeabilization of the cells wall, the blocking solution 4% BSA/PBS was added for 2 h. The primaries (diluted 1:200) were added for at least 2 h, followed by washing three times with PBS. Then the secondary antibodies (diluted 1:200) and phalloidin‐594 (diluted 1:1000) were added for 1 h and washed again three times with PBS. The nuclei were stained by conjugated DAPI (diluted 1:100) for 20 min followed by washing twice with PBS. The samples were stored in the dark at 4 °C before imaging.

### Cell Imaging via CLSM Microscopy

The stained cells were imaged using a Leica TCS SP8 microscope at lower magnification, while higher resolution imaging with subsequent deconvolution (Huygens Professional) was performed on a Leica TCS SP8 STED 3× microscope (Leica Microsystems, Germany).

### Statistical Analysis

Data points were shown as mean average with error bars indicating standard deviation (± SEM) with sample size *n* ≥ 3. Significance was calculated using one‐way analysis of variance (ANOVA) and pair comparisons using Bonferroni and Tukey's methods, and the *P* values for statistical significance are represented with stars (* *P* < 0.05, ** *P* < 0.01, *** *P* < 0.001, **** *P* < 0.0001). Statistical analysis was performed in OriginPro 2020.

## Conflict of Interest

The authors declare no conflict of interest.

## Supporting information

Supporting Information

Supplemental Movie 1

Supplemental Movie 2

Supplemental Movie 3

## Data Availability

The data that support the findings of this study are available from the corresponding author upon reasonable request.
