# Supplementary material for: Functionalized Microgel Rods Interlinked into Soft Macroporous Structures for 3D Cell Culture
Source: Adv Sci (Weinh). 2022 Jan 14;9(10):2103554. doi: 10.1002/advs.202103554 (PMC8981485; doi:10.1002/advs.202103554)
Supplement: Supplementary file 1 — Supporting Information [file ADVS-9-2103554-s004.pdf]

ADVANCED SCIENCE
Open Access

Supporting Information

for *Adv. Sci.*, DOI: 10.1002/advs.202103554

Functionalized Microgel Rods Interlinked into Soft Macroporous Structures for 3D Cell Culture

*Dirk Rommel, Matthias Mork, Sitara Vedaraman, Céline Bastard, Luis P. B. Guerzoni, Yonca Kittel, Rostislav Vinokur, Nikolai Born, Tamás Haraszti, Laura De Laporte**

Supporting Information

Functionalized Microgel Rods Interlinked into Soft Macroporous Structures for 3D Cell Culture

Dirk Rommel, Matthias Mork, Sitara Vedaraman, Céline Bastard, Luis P. B. Guerzoni, Yonca Kittel, Rostislav Vinokur, Nikolai Born, Tamás Haraszti, Laura De Laporte*

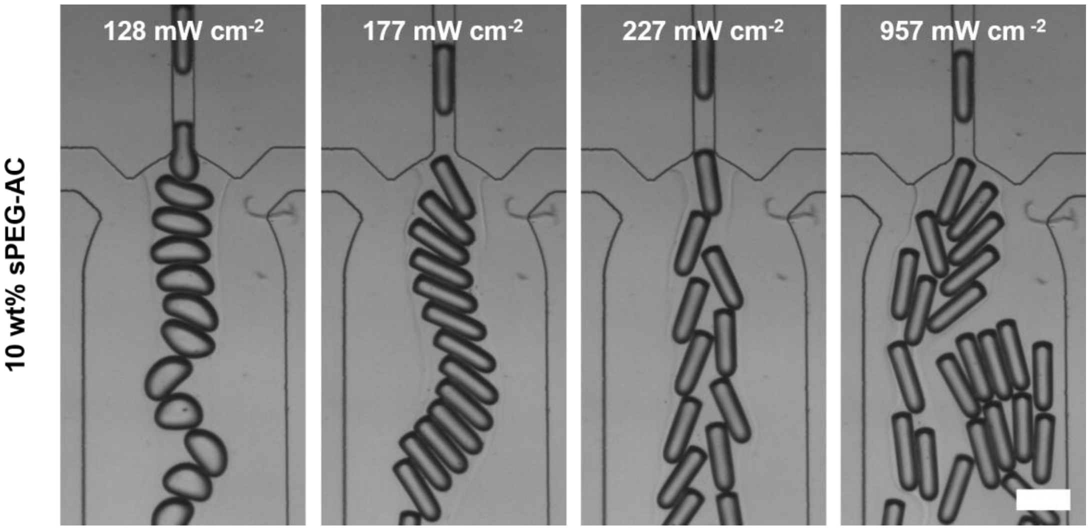

Figure S1. Flow characteristics of on-chip produced microgel rods. Bright field images of GMA-copolymerized microgel rods taken in the outlet of the microfluidic device with increasing power at λ = 365 nm from left to right (128 – 957 mW cm^{-2}) and an irradiation time ~2.3 s. Insufficiently crosslinked microgels are produced at 128 mW cm^{-2}, while from ~ 177 mW cm^{-2} on, the microgel rods retain their shape, which could lead to the assumption that the gelation is mainly completed. All flow rates are kept constant to illustrate the changing flow characteristics with increasing gelation. As gelation proceeds, the gel characteristics gradually shift away from a more liquid-like state, affecting the flow regime in the outlet. At 957 mW cm^{-2}, no further flow regime change is observed for all amine- and epoxy-functionalized microgel rod samples. Scale bar represents 200 µm for all images.

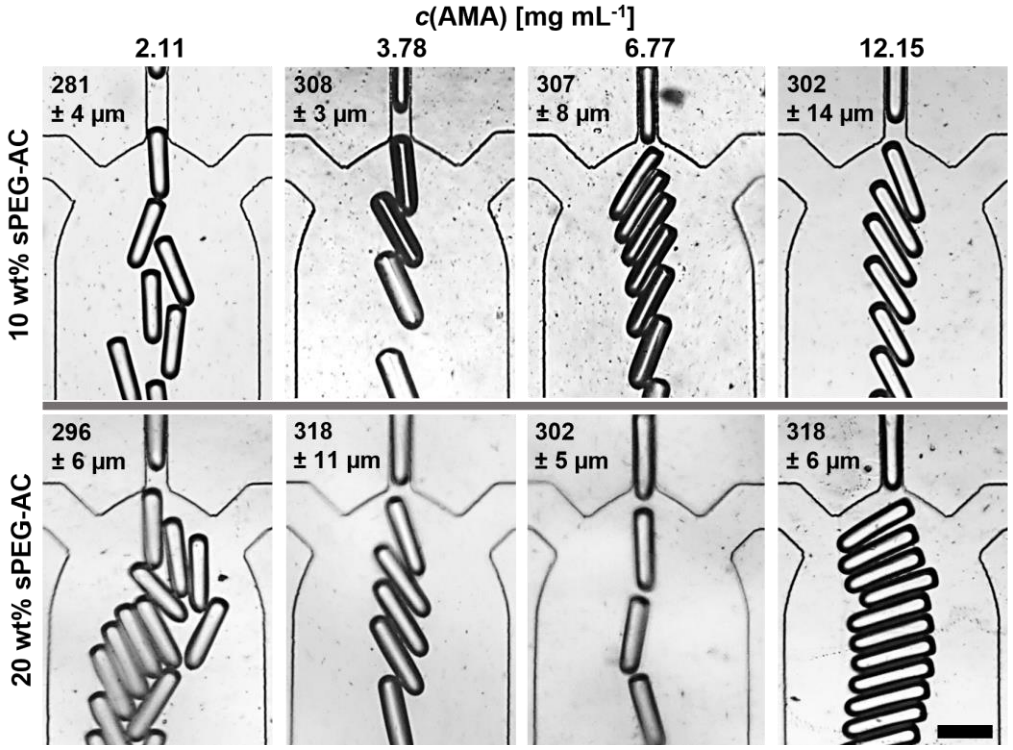

Figure S2. Amine-functionalized rods after on-chip crosslinking. Bright field images of AMA-copolymerized microgel rods taken at the outlet of the microfluidic device during production with corresponding lengths indicated in each image. The brightness and contrast are adjusted to similar values to emphasize the microgel dimensions before swelling occurs after purification. Values represent means ± SD with n=10. Scale bar represents 200 µm for all images.

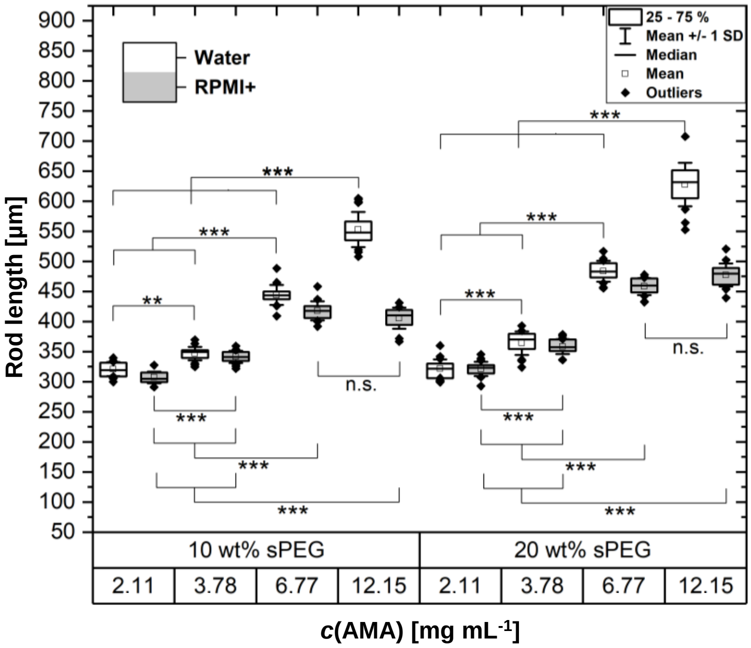

Figure S3. Dimensions of amine-functionalized microgel rods. Measured dimensions of sPEG-AC-based AMA-copolymerized microgel rods after purification in water or cell medium (RPMI+) The acquired data is displayed as a box plot, with the box extending from the 25th to 75th percentile and the whiskers reach out from 5 % to 95 % quantiles. The lines inside the boxes represent the medians, while the black points indicate means (n=20). *P*-values are calculated using one-way ANOVA with Bonferroni correction, ** $P < 0.01$, *** $P < 0.001$.

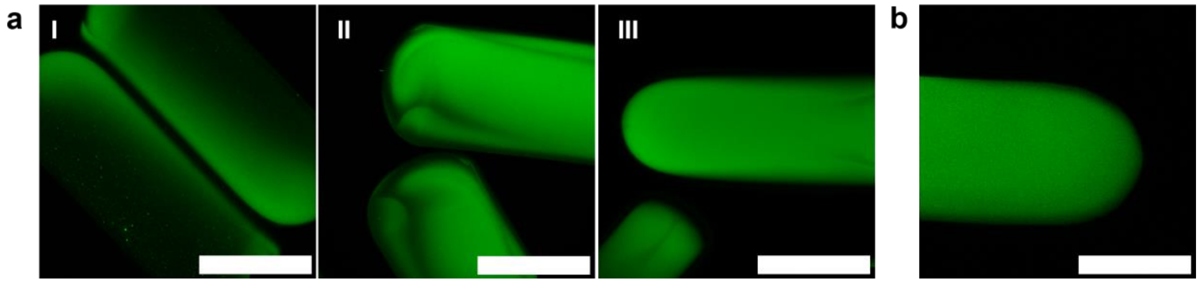

Figure S4. Determination of amine- and epoxy-functional groups via confocal microscopy. a) Confocal images of inhomogeneous FITC-signal distributions taken at the middle height of amine-functionalized microgel rods with lower AMA concentrations. I) 10 wt% sPEG-AC with c(AMA) = 2.11 mg mL^{-1}. II) 10 wt% sPEG-AC with c(AMA) = 3.78 mg mL^{-1}. III) 20 wt% sPEG-AC with c(AMA) = 2.11 mg mL^{-1}. b) Qualitative determination of epoxy functional groups via fluoresceinamine isomer I. Confocal image taken in the middle height of epoxy-functionalized 10 wt% sPEG-AC microgel rod. All scale bars represent 100 µm.

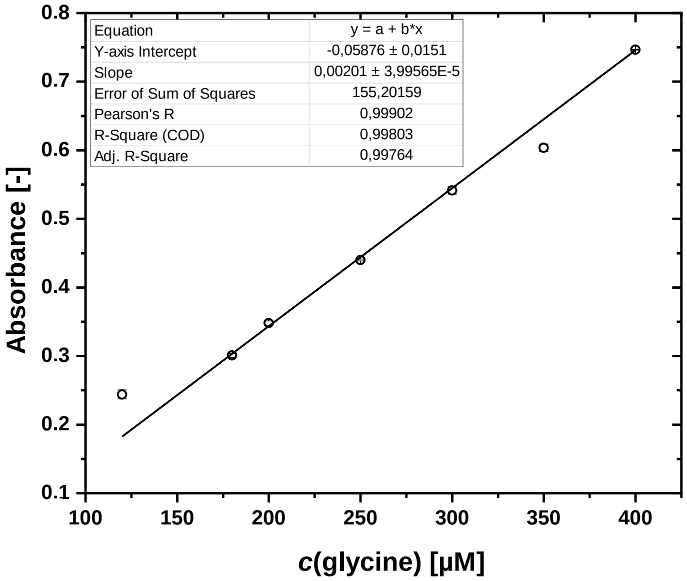

Figure S5. Ninhydrin calibration. Calibration curve of the adjusted ninhydrin assay. Solutions with known glycine concentrations are measured as standard in a threefold determination. Data points are presented as means (circles) ± SD (error bars) n=3.

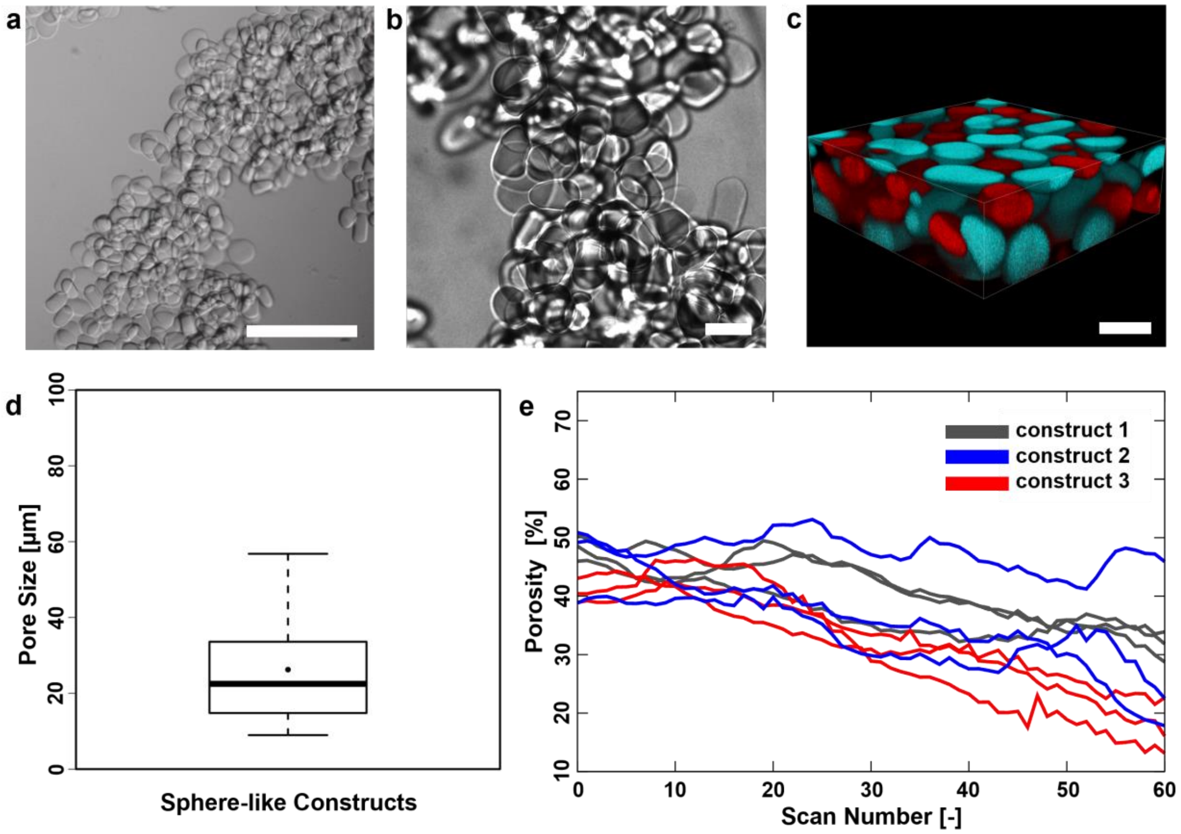

Figure S6. Sphere-like microgel assemblies (aspect ratios of 1.16 ± 0.06 with length of 143 ± 8 µm and diameter of 123 ± 4 µm for amine-functionalized microgels, and 1.42 ± 0.03 with length of 154 ± 4 µm and diameter of 109 ± 3 µm for epoxy-functionalized microgels; n=50). a) Brightfield image of a sphere-like microgel based scaffold. Poor stability of the scaffold caused by inhomogeneous microgel distributions due to inefficient microgel packing, creating weak breaking points. Scale bar represents 500 µm. b) Brightfield image of a tapering geometry between denser microgel regions found in scaffolds made from sphere-like microgels. Scale bar represents 100 µm. c) Superimposed 3D representation of a 200 µm Z-scan of a scaffold fragment with dense interlinked sphere-like microgels. Scale bar represents 100 µm. d) Quantification of confocal Z-stacks from dense construct fragments from sphere-like microgels as shown in image c. The acquired data is displayed as a box plot, with the box extending from the 25th to 75th percentile and the whiskers reach out from 5 % to 95 % quantiles. The line inside the box represents the median, while the black point indicates the mean. e) Calculated porosity of fragments from sphere-like microgels. Three different constructs were analyzed at three different locations.

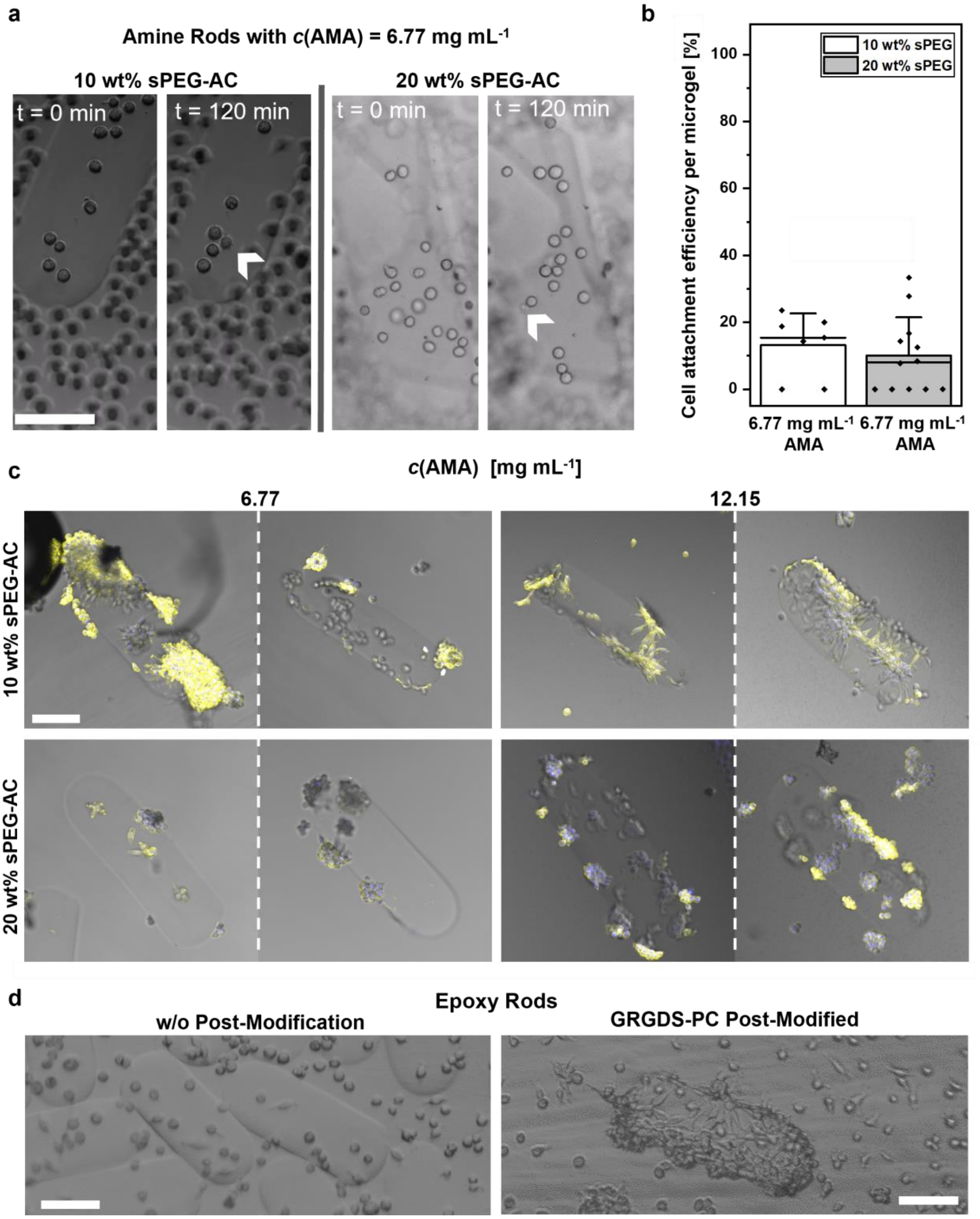

Figure S7. Cell attachment on individual microgel rods. a) Fibroblast attachment within 120 min on 10 wt% and 20 wt% sPEG-AC microgels, with an AMA concentration of 6.77 mg mL^{-1}. The scale bar represents 125 µm. Cell-microgel interaction is highlighted by white arrow heads. b) Cell attachment efficiency per microgel plot for AMA concentration of 6.77 mg mL^{-1}. The median is indicated by a black line. The data points are displayed as black rhombuses adjacent to the bars. Data presented as mean ± SD (n≥7). Data points are displayed as black rhombuses. c) Confocal microscopy images of cell growth on individual microgel rods with different AMA and sPEG concentrations at day 4. The scale bar represents 115 µm. The yellow phalloidin-iFluor signal depicts actin and the cell nuclei are visualized by blue DAPI-signal. d) Brightfield

images of cell growth on epoxy-functionalized microgels after 4 days of culturing. The scale bars represent 100 µm.

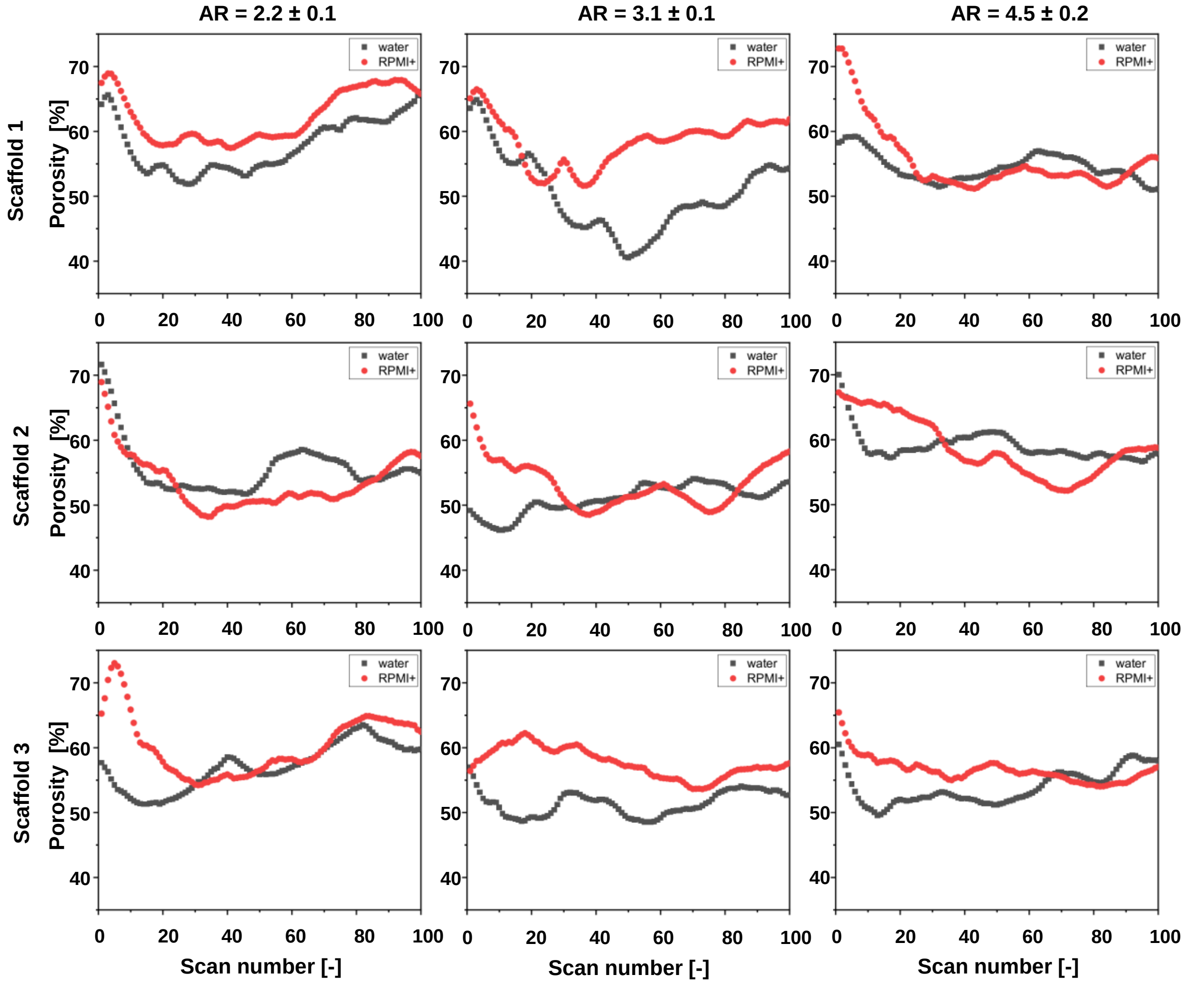

Figure S8. Calculated scaffold porosity. Individual scans of confocal Z-stacks of amine and epoxy microgel rod scaffolds (distance between scans = 5 µm), plotted against the associated calculated porosity in water and RPMI+. Epoxy-functionalized microgel rods are produced with 10 wt% sPEG-AC and different aspect ratios (2.2 ± 0.1; 3.1 ± 0.1 and 4.5 ± 0.2) (Figure 4a) and combined with amine-functionalized microgel rods (10 wt% sPEG-AC, aspect ratio: ~ 2.9 ± 0.2, 12.15 mg mL^{-1} AMA) using the same number of complementary rods (~ 1200 each). Each combination was prepared thrice.

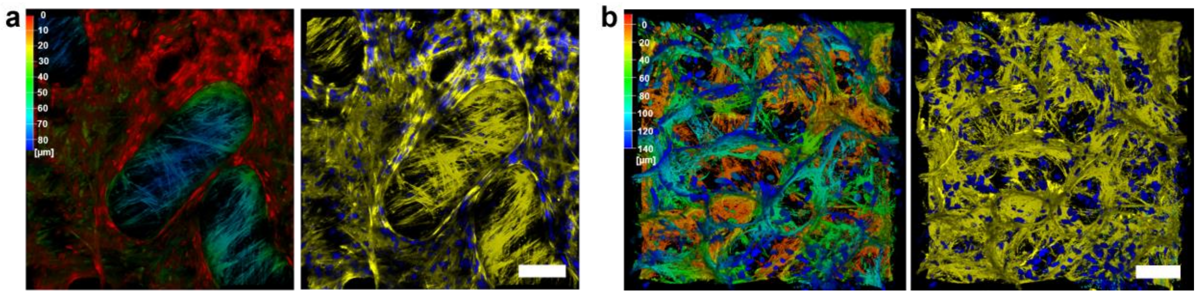

Figure S9. Depth-coded confocal imaging of human fibroblasts. a) Human fibroblasts after 12 days in culture seeded on individual AMA microgel rods (left: depth-coding of the fluorescent signal; right: superimposed 3D representation of the 100 µm Z-scan). b) Human fibroblasts

cultured inside the microgel scaffold after day 36 (left: depth-coding of the fluorescent signal; right: superimposed 3D representation of the 150 µm Z-scan). Scale bars represent 100 µm.

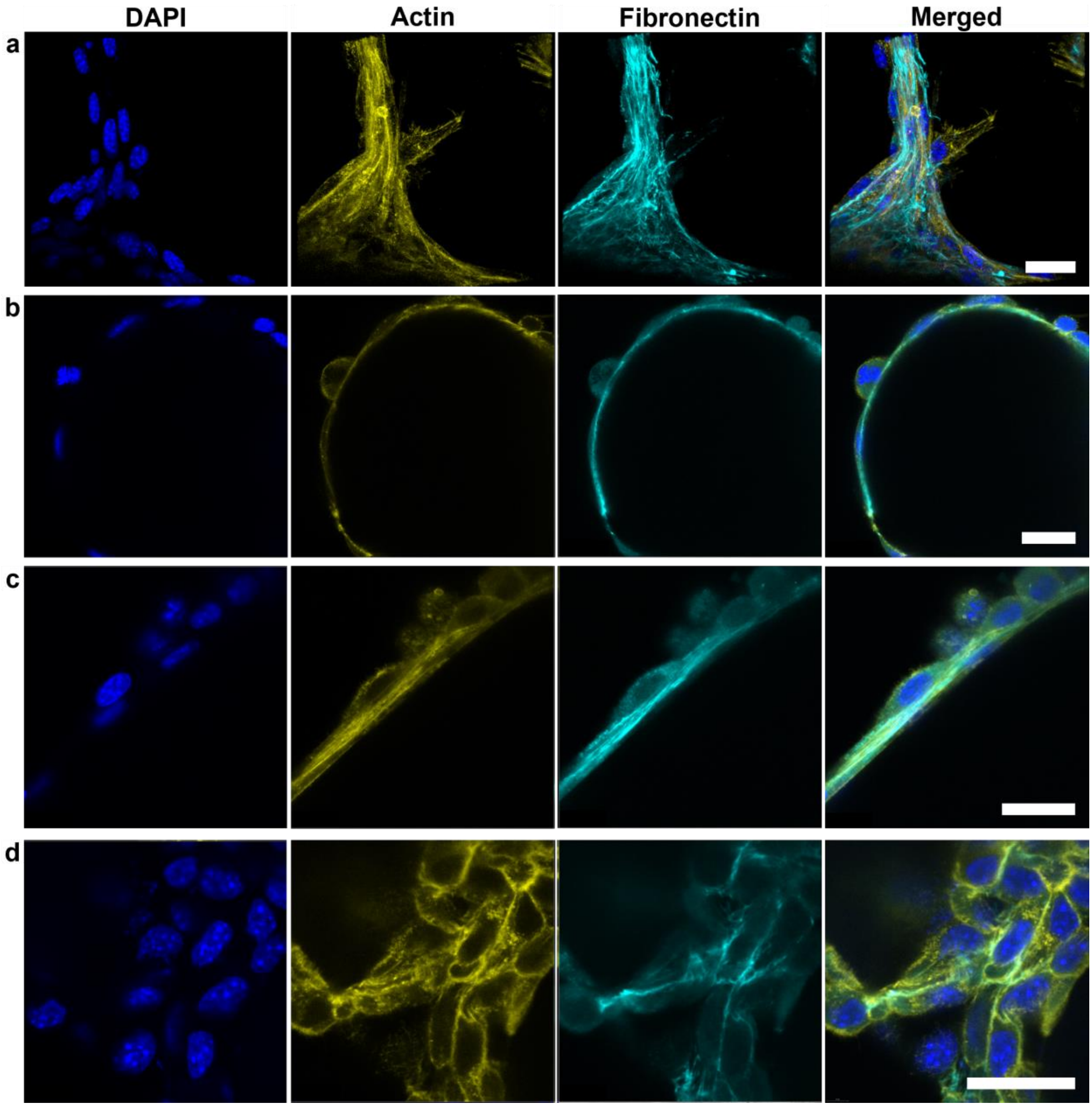

Figure S10. Confocal imaging of L929 fibroblasts displaying actin and fibronectin production after 7 days in culture seeded on microgel rod scaffolds. a) Superimposed 3D representation of the 20 µm Z-scan of cell structures in a scaffold pore. b and c) Confocal image of stretched cells covering the microgel rod and producing fibronectin that deposits at the interface between the cells and microgels. d) Cell structures formed within a scaffold pore showing fibronectin produced between cells. All scale bars represent 25 µm.

Movies

Movie S1. 3D representation of the macroporous interlinked structure. (A) Rotating 3D projection of the 500 µm confocal microscopy Z-stack of the interlinked scaffold (Fig. 3C) from epoxy-functionalized rods mixed with the same number of amine-functionalized microgel rods with the AMA concentration of 12.15 mg mL^{-1} and 10 wt% sPEG-AC. (B) Confocal Z-stack scan of the porous structure with indicated Z-values (cyan FITC-signal depicts amine-functionalized microgel rods; red signal visualizes epoxy-functionalized microgel rods via copolymerized methacryloxyethyl thiocarbamoyl rhodamine-B). Scale bar represents 200 µm for both movies.

Movie S2. Interlinked scaffold stability against strong shaking or physical stress.
(A) Shaking of an interlinked scaffold in water in real time via brightfield microscopy. Structures are prepared from epoxy-functionalized rods mixed with the same number of amine-functionalized microgel rods with the AMA concentration of 12.15 mg mL^{-1} and 10 wt% sPEG-AC. (B) Tapping of the same structure via a pipette tip in real time. Scale bar represents 500 µm for both movies.

Movie S3. Fibroblast growth inside interlinked macroporous microgel rod scaffolds. Confocal Z-stacks of L929 cells after 4 and 7 days culture shown in Fig. 6A. From left to right: Without post-modification of the epoxy-functionalized microgels, the cell network resulted in less continuous cell structures as cells only attach to the amine-functionalized microgels with GRGDS-PC. More interconnected cell structures are observed for scaffolds using epoxy microgel rods post-modified with GRGDS-PC after 4 days. Cell growth after 7 days culture in the GRGDS-PC post-modified sample. Yellow phalloidin-iFluor 594-signal depicts actin; blue DAPI-signal visualizes cell nuclei.
